# An Unusual Occurrence of Uterine Metastases in a Case of Invasive Ductal Breast Carcinoma

**DOI:** 10.7759/cureus.19820

**Published:** 2021-11-22

**Authors:** Musa Azhar, Syed Abdul Mannan Hamdani, Jhanzeb Iftikhar, Waqas Ahmad, Sajid Mushtaq, Umm-E Kalsoom Awan

**Affiliations:** 1 Medical Oncology, Shaukat Khanum Memorial Cancer Hospital and Research Centre, Lahore, PAK; 2 Radiology, Shaukat Khanum Memorial Cancer Hospital and Research Centre, Lahore, PAK; 3 Pathology, Shaukat Khanum Memorial Cancer Hospital and Research Centre, lahore, PAK

**Keywords:** gynecological metastasis, metastatic breast cancer, tamoxifen, uterine metastases, invasive ductal breast carcinoma

## Abstract

Breast cancer is the most frequent cancer in women and has a high proclivity for metastasizing, yet it seldom affects gynaecological organs. We present a case of invasive ductal carcinoma of the breast that metastasized to the uterus following initial curative treatment. Our patient was taking tamoxifen, which can induce endometrial hyperplasia and make diagnosis more complicated.

## Introduction

Breast cancer is one of the most frequent cancers in females and is treated curatively when detected early. It is the primary cause of death from cancer in females. Breast cancer has a proclivity for metastasizing, depending on the disease biology [1]. Invasive ductal carcinoma (IDC) most frequently affects the bones, liver, brain and lungs, whereas invasive lobular carcinoma (ILC) most frequently affects the adrenal glands, bone marrow, gastrointestinal and genitourinary organs [2].

Metastasis of primary breast cancer to genital organs is uncommon, with the ovaries being the most frequently involved site due to peritoneal spread. Other genital organ involvement creates a diagnostic quandary [3,4]. The instance of IDC metastasizing to gynaecological organs is presented in this report.

## Case presentation

A 49-year-old Pakistani premenopausal married lady, having hypertension, diabetes mellitus and dilated cardiomyopathy, presented with a huge breast mass with axillary extension. Physical examination showed infiltrating ulcerating retro-areolar (RA) mass approximately 10 cm large, occupying almost the whole breast. The rest of the examination was unremarkable. The mammogram showed an irregular RA mass of 9 cm with skin thickening and nipple retraction with enlarged lymph nodes. Baseline metastatic workup, including computed tomography (CT) of chest abdomen pelvis and bone scan, was negative for metastasis. Histopathology of trucut biopsy from left breast showed IDC grade II. Immunohistochemistry stains showed oestrogen receptor (ER) 70%, progesterone receptor (PR) 40%, human epidermal growth factor receptor 2 (HER2) was negative and Ki-67 of 10%-15%. The axillary lymph node biopsy was positive for metastasis.

**Figure 1:**
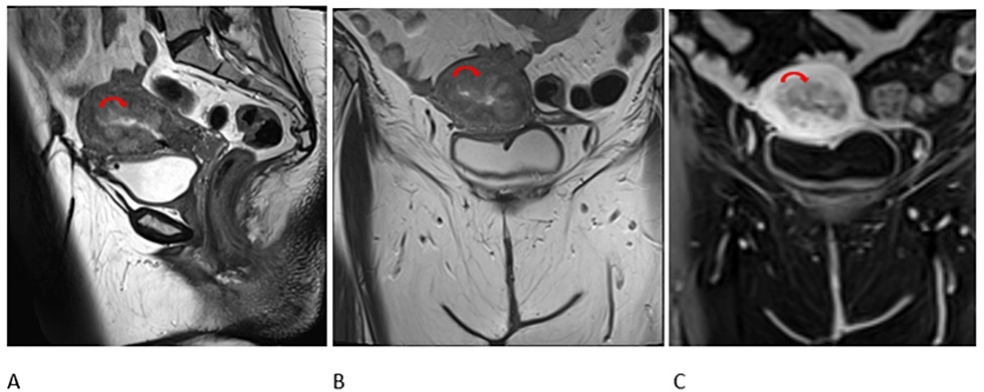
Sagittal T2 (A), coronal T2 (B) and post-contrast T1 (C) MRI images through the pelvis show irregular lobular mass lesions around the endometrial cavity and extending into the myometrium (red arrows).

**Figure 2:**
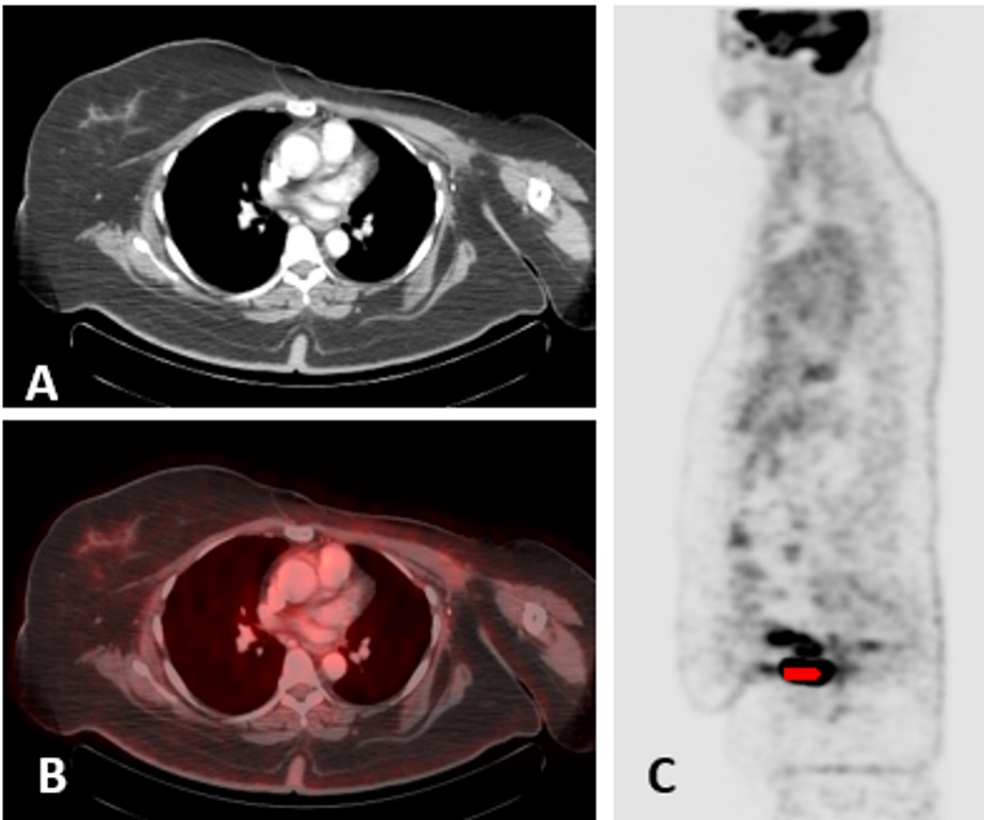
Axial CT/PET-CT images (A, B) show changes of left mastectomy without any local recurrence. Hypermetabolic endometrial lesions were noted on sagittal PET-only image (C) indicated by arrowhead.

**Figure 3:**
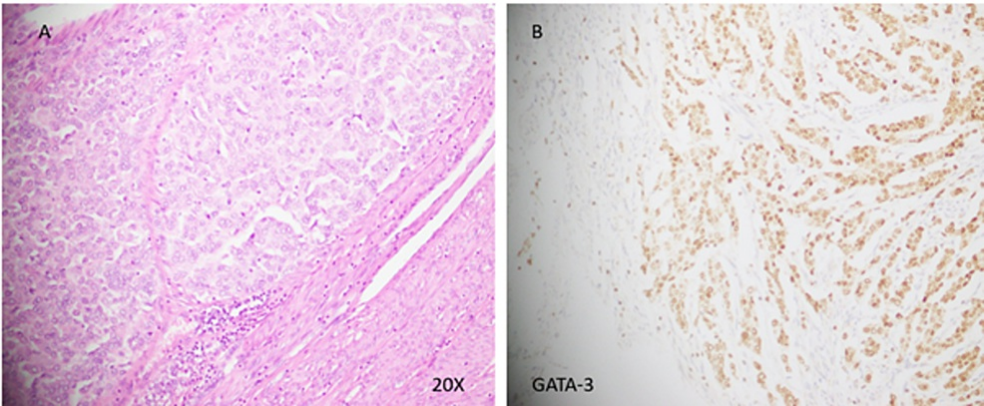
(A) Section from myometrium shows a tumour comprised sheets and trabeculae of moderately atypical tumour cells with rounded nuclei and prominent nucleoli. (B) These tumour cells are positive for GATA-3 immunohistochemical stain, which indicated the primary tumour of breast origin.

As per the multidisciplinary team (MDT) meeting recommendations, neoadjuvant chemotherapy followed by modified radical mastectomy (MRM) and radiotherapy/endocrine therapy. She received adriamycin, cyclophosphamide and paclitaxel-based chemotherapy followed by MRM and axillary lymph node dissection; histopathology after mastectomy showed residual IDC 5.5 cm, with 12 lymph nodes positive for metastatic carcinoma with extracapsular spread. She was given radiotherapy (40Gy in 15 fractions) to the left supraclavicular fossa and left chest wall. Eight months after starting tamoxifen, she reported mild per vaginal bleeding. The transvaginal scan showed a 10-mm endometrial strip with mild free fluid. MRI pelvis delineated irregular lobulated lesion in the uterus (Figures 1A-1C). After gynaecological consultation, dilatation and curettage were done. Histopathology showed poorly differentiated carcinoma, raising suspicion of breast metastasis versus primary uterine carcinoma. PET-CT scan showed hypermetabolic endometrial lesions without any local recurrence (Figures 2A-2C). The case was re-discussed in the weekly gynaecology MDT meeting; the patient underwent abdominal hysterectomy and bilateral salpingo-oophorectomy. Final pathology showed poorly differentiated carcinoma of endometrium, tumour involved greater than half of the myometrium, and cervical stroma; the lymphovascular invasion was seen consistent with breast primary. Stains showed CK: Positive, p53: Positive, p63: Negative, Oestrogen receptors: Positive, GATA-III: Diffuse positive, p16: Focal positive, PAX-8: Focal positive, Mammaglobin and GCDFP-15 were also positive (Figures 3A, 3B).

Omental tissue had evidence of dystrophic calcification. Bilateral parametria were free of tumour. Bilateral ovaries and fallopian tubes were also free of tumour.​​​​​​​ 

She has been commenced on letrozole and ribociclib and is doing well for six months.

## Discussion

We have reported a case of IDC metastasizing to the uterus, which presented eight months of initial treatment with per vaginal bleeding while on tamoxifen. The unusual, isolated metastases to the endometrial lining make this presentation unique. Genital metastases are rare entities associated with breast cancer; mostly, ovaries are the most affected organs. Isolated uterine metastases are caused by hematogeneous spread [5]. Endometrial involvement can be asymptomatic or present as abnormal uterine bleeding (AUB), as in our case [6].

Tamoxifen is vital in treating hormone-positive breast cancer, with a significant recurrence risk reduction and survival benefit. But it is associated with the risk of endometrial hyperplasia and malignant transformation in 2.7% of patients due to its agnostic effect on the endometrial lining [7-9]. As this patient was on tamoxifen, AUB posed a diagnostic dilemma.

A comprehensive is a review done by Ayesha et al. from 1984 to 2017 reported 25 cases of endometrial metastases from breast cancer, 13 from ILC, nine from IDC, one apocrine, one metaplastic and one mixed [10].

The uterine metastases from breast cancer can pose a diagnostic dilemma; specific immunohistochemical stains shall be performed to differentiate uterine metastases from primary uterine carcinoma [11].

The ideal approach to isolated uterine metastases is still unknown; case reports are favouring treating with chemotherapy, but in case of a diagnostic dilemma, surgical intervention can be both diagnostic and therapeutic [10,12,13]. The prognosis following chemotherapy or surgery in such a case is still unknown [14]; our patient has been disease-free since surgery.

## Conclusions

In conclusion, AUB in patients with breast cancer on hormonal therapy should alarm physicians to look for primary uterine malignancy. While keeping uterine metastasis in the differential diagnosis is also crucial for early intervention and appropriate management. A keen histopathological analysis can help in reaching the correct diagnosis.

## References

[1] Riggio AI, Varley KE, Welm AL (2021). The lingering mysteries of metastatic recurrence in breast cancer. Br J Cancer.

[2] Chikarmane SA, Tirumani SH, Howard SA, Jagannathan JP, DiPiro PJ (2015). Metastatic patterns of breast cancer subtypes: what radiologists should know in the era of personalized cancer medicine. Clin Radiol.

[3] Mazur MT, Hsueh S, Gersell DJ (1984). Metastases to the female genital tract: analysis of 325 cases. Cancer.

[4] Gasparri ML, Taghavi K, Fiacco E (2019). Risk-reducing bilateral salpingo-oophorectomy for BRCA mutation carriers and hormonal replacement therapy: if it should rain, better a drizzle than a storm. Medicina (Kaunas).

[5] Arslan D, Tural D, Tatlı AM, Akar E, Uysal M, Erdoğan G (2013). Isolated uterine metastasis of invasive ductal carcinoma. Case Rep Oncol Med.

[6] Rahmani M, Nili F, Tabibian E (2018). Endometrial metastasis from ductal breast carcinoma: a case report with literature review. Am J Case Rep.

[7] Fisher B, Costantino JP, Redmond CK, Fisher ER, Wickerham DL, Cronin WM (1994). Endometrial cancer in tamoxifen-treated breast cancer patients: findings from the National Surgical Adjuvant Breast and Bowel Project (NSABP) B-14. J Natl Cancer Inst.

[8] Famoriyo A, Sawant S, Banfield PJ (2004). Abnormal uterine bleeding as a presentation of metastatic breast disease in a patient with advanced breast cancer on tamoxifen therapy. Arch Gynecol Obstet.

[9] Piura B, Yanai-Inbar I, Rabinovich A, Zalmanov S, Goldstein J (1999). Abnormal uterine bleeding as a presenting sign of metastases to the uterine corpus, cervix and vagina in a breast cancer patient on tamoxifen therapy. Eur J Obstet Gynecol Reprod.

[10] Akhtar A, Ratra A, Puckett Y, Sheikh AB, Ronaghan CA (2017). Synchronous uterine metastases from breast cancer: case study and literature review. Cureus.

[11] Braxton DR, Cohen C, Siddiqui MT (2015). Utility of GATA3 immunohistochemistry for diagnosis of metastatic breast carcinoma in cytology specimens. Diagn Cytopathol.

[12] Huo Z, Gao Y, Zuo W, Zheng G, Kong R (2015). Metastases of basal-like breast invasive ductal carcinoma to the endometrium: a case report and review of the literature. Thorac Cancer.

[13] Çift T, Aslan B, Bulut B, İlvan Ş (2016). Unusual uterine metastasis of invasive ductal carcinoma: a case report. Turk J Obstet Gynecol.

[14] Karvouni E, Papakonstantinou K, Dimopoulou C, Kairi-Vassilatou E, Hasiakos D, Gennatas CG, Kondi-Paphiti A (2009). Abnormal uterine bleeding as a presentation of metastatic breast disease in a patient with advanced breast cancer. Arch Gynecol Obstet.

